# Points in the Treatment of Fistula-In-Ano

**Published:** 1908-08-29

**Authors:** J. Bernard Dawson

**Affiliations:** Late House Surgeon to St. Mark's Hospital for Diseases of the Rectum.


### SPECIAL ARTICLE.

### POINTS IN THE TREATMENT OF FISTULA-IN-ANO.

By J. BERNARD DAWSON, M.B., B.S., F.R.C.S., L.R.C.P., Late House Surgeon to St. Mark's Hospital for Diseases of the Rectum.

THE treatment of fistula naturally resolves itself into palliative and operative. In the main palliative treatment is useless and waste of time; there are, however, circumstances which render it the only method available or justifiable. But in a patient whose general health and age do not contra-indicate operative measures no reluctance on the patient's part should hinder the surgeon from using all the means in his power to persuade the former to submit to operation.

The two factors which make operative measures unjustifiable are extreme age and advanced tuberculosis of the lungs or other important viscera. Extreme age renders the administration of an anæsthetic, the essential confinement to bed, and the operative shock dangerous. Advanced tuberculosis renders the anæsthetic dangerous and the hope of cure remote. In slight or latent phthisis, however, the removal of a perineal focus of tubercle bacilli has an undoubted beneficial effect upon the pulmonary invasion.

Cases of extreme prostration, emaciation, or asthenia can be omitted, since these are conditions which can be so improved by treatment as to render operation at a later date advisable. In diabetes and albuminuria operation is not contra-indicated, and many cases are on record where operation has been perfectly successful, notwithstanding the presence of sugar or albumen in the urine. The presence of uræmia or of a diabetic diathesis approaching coma is naturally a bar to any surgical procedure.

Palliative treatment consists of:—

1. Dilatation of the external orifice to promote better drainage. This can be accomplished by bougies, expansile tents, or nicking with a scalpel.
2. Daily attention to the anal region; thorough cleansing with soap and water, followed by a mild antiseptic, such as 2 per cent. carbolic acid, and attention to any excoriation or pruritus of the skin around.
3. Irrigation of the rectum after defæcation, so that the internal opening, if present, shall be free from contact with fæcal fragments. For this a mild unirritating antiseptic should be used.
4. Injection of antiseptic along the track of the fistula. For this purpose a glass sinus nozzle attached to a tube and funnel answers best. Any of the following solutions are useful:—Hydrogen peroxide, vol. 20 per cent.; lotio acidi carbolici, 2 per cent.; lotio acidi boracis; emulsio iodoformi, 5 per cent.
5. Exhibition of cod-liver oil, malt extract, iron, strychnine or nux vomica, syrup of hypophosphites or syrupus ferri phosphatis cum quininâ et strychninâ; change of air, rest, or a sea voyage; prohibition of cycling, rowing, riding, or excessive walking.
6. Regulation of the bowels. For this purpose $\frac{1}{8}$ gr. of calomel three times a day is very useful, or a drachm of sulphate of soda in water on arising, or the following formula:—

| | | |
|---|---|---|
| ℞ | Pulveris Jalapæ Compositi ... ... | gr. xv. |
| | Pulveris Sennæ Alexandrinæ ... | gr. xv. |
| | Sulphuris Præcipitati ... ... | gr. xx. |
| | Aquæ Destillatæ ... ... ... | ℥j. |
| | Theriacæ opt. ... ... ... | ad ʒj. |

Misce. Fiat Confectio.
Signa: A teaspoonful to be taken at bedtime.

In operating on cases of fistula there are many important details, attention to which makes for the best results. If possible, the patient should allow two days for preparation for the operation. On the first night he should be given pilulæ colocynthidis et hyoscyami gr. vi., to be followed next morning by a large simple enema. On the second night he should take a hot bath, and upon retiring a tablespoonful of castor oil. This is again followed in the morning by a large simple enema, after which the patient takes another bath.

This apparently drastic treatment is important, for these patients are usually constipated, because of the pain experienced on defæcation. Moreover, anyone who has experienced the discomfort of an emergency operation upon the rectum will realise the advantage of dealing with an empty bowel. This method of preparation will be found useful for all rectal operations. The perineum need not be shaved except in particularly hirsute men, for the hair does not hinder healing, whereas the short, stubby growth consequent upon shaving is a source of much irritation to the patient.

The anæsthetic is a matter of choice; ether, though safer than chloroform, renders the part fuller of blood and tends to aggravate the laryngeal spasm so often experienced in rectal work. The writer's personal preference is for a mixture of $E_2C$, given upon an open mask. Medullary anæsthesia, whilst quite efficient, has a great disadvantage, in that it does not render the patient unconscious to the discomfort of the lithotomy position and to the natural abhorrence of surgical interference in the rectal area. Especially is this so in female patients.

The local preparation should be as thorough as in other parts of the body. Ether soap followed by biniodide of mercury 1-500 in methylated spirit for the skin, and lysol, ʒj to the pint, for the mucous membrane are satisfactory. The rectum should be thoroughly washed out with the lysol solution.

If a line be drawn transversely through the anus and the main external opening is found to be behind this line, then, in the great majority of cases, the internal opening is in the mid-line dorsally, immediately above the external sphincter. The fistula may be a simple dorsal one or, if the external opening be situated laterally, a semi-horseshoe one. The reason of this is that fistulæ commonly originate from a lesion of the mucous membrane at this point, a point exposed to the greatest friction and pressure upon the passage of a hard fæcal mass. If the opening be anterior to the transverse line, then the internal opening may be either in line with it or in the mid-line anteriorly, the former being the most common.

Fistulæ with two internal openings are rare, but those with several external openings are very common. The secondary external openings may be the mouths of branches from one main track, or, if situated on the opposite side of the anus, evidence of a double horse-shoe fistula, in which two semi-lunar tracks lead from one internal orifice situated in the mid-line dorsally.

Where the track is straight, incision is easily done by cutting with a bistoury along a probe-pointed director. When, however, the track is curved, some manipulation is needed to avoid cutting the sphincter obliquely, a manœuvre which is bad practice, since it may lead to subsequent incontinence. In such a case two methods of equal value are practised. One is to make a side cut in the perineum so as to bring the director at right angles to the sphincter, or to project the point of the director at the height of the convexity of the curve, and then cut on to it, so as to bring it through the skin. The outer half of the track is then slit up, and the instrument passed again through to the internal opening, and the incision of the fistula completed. If the internal opening be situated above the lower border of the internal sphincter, it is a safe rule never to cut more than the lower fibres of the muscle; otherwise there is danger of incompetence occuring later.

Another device, known as Salmon's back cut, consists in incising freely into the brawny tissue lying around the external opening of the fistula. This is useful for the subsequent dressing, and promotes healing, since it prevents any tendency to reformation of the track. The overhanging edge of the incised track should be freely cut away, even to an apparent sacrifice of healthy tissue. The object is to have a plane raw surface, so that healing will take place from the edges. The granulation tissue lining the track must be thoroughly curetted away with a sharp spoon, and some surgeons advise that the fibrous tissue underneath should be freely incised longitudinally with a scalpel. Branches and side-tracks must be thoroughly sought for, and can be seen, if a good light is used, after the granulation tissue is removed and bleeding arrested.

A word of warning is needed as to the ease with which an inexperienced surgeon can push a probe into the fatty tissue around the rectum, and so manufacture tracks. A properly manipulated director should pass easily and without pressure into accessory sinuses.

In the majority of cases the external sphincter will have been divided if the above details have been carried out; this is essential if proper rest to the part is to be attained. However, some small fistulæ do not include the sphincter, the internal opening being below or in the midst of the muscular fibres. When this is the case, complete division is unnecessary and a useless mutilation.

The introduction of a morphia suppository after operation is well replaced by opium in some form by the mouth after the patient has recovered from the anæsthetic. The practice of St. Mark's was to give, if necessary, twenty minims of tincture of opium about three hours after the operation. If this is insufficient to relieve pain, $\frac{1}{4}$ gr. of morphia with 1-120 gr. of atropine was given hypodermically. This plan was found most effectual, and obviated the introduction of a foreign body into the rectum or the unnecessary administration of an opiate.

The dressing at the time of operation consists of gauze or wool packed into the wound, over which a graduated compress of sterilised wool is secured by a T-bandage. If necessary this outer wool is changed when soiled or bloodstained. The patient is kept on a low diet of milk and beef tea for four days. On the fourth night after operation one ounce of castor oil is given, followed by a bath after the bowels have acted. No trouble will be experienced from four days' constipation, if the initial preparation has been sufficiently vigorous. After this the patient is dressed every four hours, three times or twice a day, depending upon the size of the wound and the amount of discharge. The dressing consists of flat squares of cotton wool, moist and sterilised with perchloride of mercury lotion 1-2,000. This is wrapped round a probe and passed along the wound into the rectum. For this purpose a special probe is very useful, consisting simply of six inches of knitting needle, the size of a quill, with rounded ends. There should be no shoulders or waist in the shaft of this instrument, otherwise the dressing will catch and be withdrawn with the probe.

Once a day the finger should be inserted into the rectum to make sure that healing is satisfactory and that no pockets form under tags of mucous membrane. Attention must also be paid to healing on the surface, which produces bridges of tissue with new tracks beneath. The patient must be kept absolutely in bed, except for his morning and evening bath. These baths are a most important aid to convalescence after operation for fistula-in-ano. They keep the parts clean in a way which the most careful irrigation cannot. The addition of boracic acid or carbolic acid to the bath is helpful.

When the wound is sound, or at most there is only a small superficial area of granulation tissue, the patient may be allowed up; but no walking about must be permitted until the healing is quite complete.

The aim of the surgeon should be to turn the fistula under his care, not into a fissure, but into a flat ulcer—that is, to pare away the edges of the incised fistula so that the resulting wound is a flat surface and not of the nature of a crevice. If this is done, healing will be straightforward, and dressing simple and painless. Attention to operative details, unremitting care, frequent dressing, and constant baths spell success in the treatment of this vexatious and neglected condition; whilst faulty technique, neglected dressing, infrequent digital examination, and delay spell disaster both to the patient and to the professional reputation.

